# Elevated SIRT2 of serum exosomes is positively correlated with diagnosis of acute ischemic stroke patients

**DOI:** 10.1186/s12883-023-03348-7

**Published:** 2023-09-08

**Authors:** Wenmei Lu, Duanlu Hou, Xin Chen, Ping Zhong, Xueyuan Liu, Danhong Wu

**Affiliations:** 1 Department of Neurology, Shanghai Fifth People’s Hospital, Fudan University, 801 Heqing Road, Shanghai, China; 2 Department of Geriatrics, Shanghai East Hospital, Tongji University School of Medicine, 150 Jimo Road, Shanghai, China; 3 Department of Neurology, Shanghai Yangpu District Shidong Hospital, 999 Shiguang Road, Shanghai, China; 4 Department of Neurology, Shanghai Tenth People’s Hospital, Tongji University School of Medicine, 301 Middle Yanchang Road, Shanghai, China

**Keywords:** SIRT2, Exosomes, Acute ischemic stroke, Blood biomarker, Diagnosis

## Abstract

**Background:**

Silent Information Regulator 2 (SIRT2) protein inhibition has been shown to play a neuroprotective role in acute ischemic stroke (AIS) in mice. However, its role in AIS patients has not been fully understood. In this study, we aimed to analyze SIRT2 protein expression in serum exosomes of AIS and non-AIS patients, and evaluate its potential role in diagnosis and prognosis of AIS.

**Methods:**

Serum exosomes from 75 non-AIS subjects and 75 AIS patients were isolated. The SIRT2 protein levels in exosomes were analyzed using enzyme linked immunosorbent assay (ELISA). The National Institutes of Health Stroke Scale (NIHSS) was used to evaluate the severity of the disease. The modified Rankin Scale (mRS) was employed to assess the functional outcomes of the patients at 3-months following stroke onset.

**Results:**

The SIRT2 protein concentration of serum exosomes were higher in AIS patients than non-AIS patients (p < 0.001). Furthermore, the receiver operative characteristic curve (ROC) demonstrated that higher serum exosome SIRT2 could differentiate AIS patients from non-AIS patients with a sensitivity of 81.3% and a specificity of 75.3%. The area under the curve was 0.838 (95% CI: 0.775, 0.902). Additionally, higher SIRT2 concentration of serum exosomes were associated with NIHSS ≥ 4 (p < 0.001) and mRS ≥ 3 (p = 0.025) in AIS patients. The ROC analysis showed SIRT2 could discriminate stroke with NIHSS ≥ 4 from mild stroke (NIHSS < 4) with a sensitivity of 75.0% and a specificity of 69.6%. The area under the curve was 0.771 (95% CI: 0.661,0.881). Similarly, the test showed SIRT2 could differentiate between AIS patients with mRS ≥ 3 from those with mRS < 3 with a sensitivity of 78.3% and a specificity of 51.9%. The area under the curve was 0.663 (95% CI: 0.531,0.796). The logistic regression analysis revealed that SIRT2 concentration in serum exosomes can independently predict the diagnosis of AIS (odd ratio = 1.394, 95%CI 1.231–1.577, p < 0.001) and higher NIHSS scores (≥ 4) (odd ratio = 1.258, 95%CI 1.084–1.460, p = 0.002). However, it could not independently predict the prognosis of AIS (odd ratio = 1.065, 95%CI 0.983–1.154, p = 0.125).

**Conclusion:**

The elevation of SIRT2 in serum exosomes may be a valuable biomarker of AIS, which may be a potential diagnostic tool to facilitate decision making for AIS patients.

**Supplementary Information:**

The online version contains supplementary material available at 10.1186/s12883-023-03348-7.

## Introduction

Ischemic stroke is the leading cause of death and disability in China. As a result of the occlusion of cerebral blood vessels, patients may suffer from hemiplegia paralysis, aphasia, coma, and even death [1]. The risk factors associated with stroke are numerous, ranging from modifiable factors, such as obesity, diabetes mellitus, smoking, and high blood pressure to non-modifiable factors such as aging [2]. In recent years, computed tomography (CT) and/or magnetic resonance imaging (MRI) have become valuable diagnostic tools to diagnose ischemic stroke [3, 4]. Moreover, a recent study has identified an inverse association between ischemic stroke risk and circulation total bilirubin [5]. Additionally, another study has revealed the increased apolipoprotein B, low-density lipoprotein (LDL), and triglycerides are related to a higher risk of ischemic stroke [6]. Furthermore, the use of proprotein convertase subtilisin/kexin type 9 (PCSK9) inhibitors could prevent stroke by reducing LDL-C [7]. Overall, the detection of effective blood biomarkers is emerging as a valuable predictive tool to guide therapeutic strategies for stroke patients. Researchers are persistently exploring new diagnostic and treatment options to improve the prognosis for patients suffering from ischemic stroke in China.

Silent Information Regulator 2 (SIRT2), a member of the Sirtuins family, is an NAD^+^-dependent lysine deacetylase [8, 9], which plays a vital role in physical and pathological processes, such as the induction of telomeric silencing, cell mobility, suppression of tumorigenesis, and regulation of longevity [10, 11]. Recent studies have shown that SIRT2 is involved in the pathogenesis of neurological disorders, including neurodegenerative diseases, stroke, and tumors of the central nervous system. For example, SIRT2 deacetylates tubulin to regulate myelinogenesis and oligodendroglia differentiation [9, 12]. The SIRT2 inhibitor AK-7 has been shown to ameliorate the deterioration of Parkinson’s disease and Huntington’s disease, but not amyotrophic lateral sclerosis [13, 14]. In neuroblastoma, SIRT2 has been reported to suppress tumor growth by sumoylation modification [15]. In our previous study, SIRT2 inhibitor AK7 was found to decrease the infarct area, and improved behavior in mouse stroke models by activating the p38 Mitogen-Activated Protein Kinase (MAPK) signal pathway [16]. However, there is still little known about the association between SIRT2 and neurological disorders.

Blood biomarkers have shown promise in aiding the diagnosis and treatment of various diseases. In particular, the concentration of serum SIRT2 has been found to potentially differentiate Parkinson’s disease from Parkinsonism syndromes [17]. However, it is important to note that levels of biomarkers in the blood could not reflect their levels in the brain due to the blood-brain barrier. Recent reports have identified that multiple neural cells could secret exosomes. Neural cells derived exosomes could be detected in the cerebrospinal fluid and plasma, making them a more accurate representation of brain activity [18–20]. Exosomes contain a variety of contents including proteins, lipids, nucleic acids, organelle fragments, and metabolites, which can be used as potential diagnostic tools [21]. In this study, patients with acute cerebral infarction were followed for 90 days to investigate the relationship between serum exosome SIRT2 content and acute stage severity of cerebral infarction and 90-day prognosis. We aimed to explore the role of levels of SIRT2 in serum exosomes in the diagnosis and prognosis of acute ischemic stroke patients.

## Materials and methods

### Patients

In this prospective study, a total of 75 patients were recruited from the Neurology Department of the fifth hospital affiliated to Fudan University during the period of 2020 to 2022. The patients who showed symptoms of acute ischemic stroke within 24-hour time frame was consecutively included in the study. The diagnosis of acute ischemic stroke was confirmed by typical symptoms, such as hemiplegia, aphasia, and facial paralysis, as well as brain computed tomography (CT) and/or magnetic resonance imaging (MRI). To maintain the integrity of the study, patients with a history or presence of cancers, severe inflammation, severe hepatic or renal diseases were excluded from the study. All patients were followed up to 90 days or death, and the modified Rankin Scale (mRS) was used to evaluate the 90-day outcome of patients.

### Control group

A total of 75 subjects without cerebrovascular diseases from our hospital were included as the control group. However, these subjects had a number of high-risk factors of cerebrovascular diseases, including hypertension, diabetes, smoking, and / or hyperlipemia. To ensure the reliability of the results, subjects with a history of ischemic or hemorrhagic stroke, cancers, severe inflammation, severe hepatic or renal diseases were excluded. Written informed consent was collected from all subjects.

### Data collection

After admission, demographic characters (age, sex), and vascular risk factors (drinking, smoking, diabetes, hypertension, total cholesterol, triglyceride, low-density lipoprotein, fasting blood glucose, creatinine, and uric acid) were all recorded. Stroke severity was evaluated by a trained neurologist using the National Institutes of Health Stroke Scale (NIHSS) with a total score ranging from 0 to 42 at the time of hospital admission.

### Follow-up

Acute ischemic stroke patients were asked to make face-to-face follow-up visits at the neurology clinic 90 days after the onset of stroke symptoms or until the point of death. The 90-day functional outcome after stroke attack was analyzed by a trained neurologist using the modified Rankin Scale (mRS) with a total score ranging from 0 to 6. A mRS score lower than 3 was considered a desirable outcome.

### Blood collection and processing

Blood samples were collected within 24 h from stroke symptoms onset in centrifuge tubes without anticoagulant and the serum was rapidly separated and stored at -80℃. Serum samples were thawed in a 25℃ bath and placed on ice. Serum samples were centrifuged at 2000×g for 30 min and the supernatant transferred to a new tube. The Total Exosome Isolation reagent (ThermoFisher Scientific, #4,478,360) of 0.2 volume of the serum sample was added and mixed with the serum. After incubation on ice for 30 min, the mixture was centrifuged at 10,000 ×g for 10 min at room temperature, and the pellet was obtained for further experiments.

### Protein concentration assay by ELISA

The pellet was solubilized in chilled protein extraction buffer provided by the Human SIRT2 SimpleStep ELISA Kit (Abcam, #ab227895). The concentration of protein samples was analyzed by following the procedures provided by the manufacturer. Protein samples and capture/detector antibody cocktails were consecutively added to microplate strips. After incubation, each well was washed 3 times. TMB substrate was then added to each well and incubated in the dark. Finally, the stop solution was added to stop the reaction and the OD value of each sample was recorded at 450 nm. A standard curve was constructed and concentrations of the protein samples were calculated.

### Western blot analysis

The serum exosomes pellet supernatant protein was loaded for electrophoresis on 10% sodium dodecyl sulfate polyacrylamide gel electrophoresis (SDS-PAGE) and then transferred to a 0.45-µm polyvinylidene fluoride (PVDF) membrane. The PVDF membrane was blocked with 5% non-fat milk for 1 h at room temperature and then incubated with primary antibody at 4℃ overnight. After immersed in the Horseradish Peroxidase (HRP) - conjugated secondary antibody, the target bands were detected by enhanced chemiluminescence (ECL) liquid. And the primary antibodies were purchased from abcam, including CD63 antibody (#ab217345, abcam, Cambridge, UK), and CD81 antibody (#ab79559, abcam, Cambridge, UK).

### Statistical analysis

The statistical analysis for this study was conducted using two software programs: Statistical Product and Service Solutions (SPSS (IBM, Chicago, USA)) and GraphPad Prism (GraphPad Software lnc., San Diego, USA). The baseline characteristics of the study participants were presented as either mean ± standard deviation (SD) or median value with an interquartile range (IQR) for continuous variables, and as a percentage (%) for categorical variables. To compare between groups, the Mann-Whitney U test or independent sample t-test was used for continuous variables, While the Chi-square test was used for categorical variables. Binary Logistic regression analysis was employed to identify independent factors that contribute to the development of AIS, specifically when the p-value of these variables was less than 0.1. In terms of sensitivity and specificity, the receiver operating characteristic (ROC) curves were used. To determine the cutoff and associated sensitivity and specificity values, the max Youden index (sensitivity + specificity-1) was utilized. Additionally, the area under the curve (AUC) was used to compare the predictability of proteins. Furthermore, restricted cubic spline was used to examine the correlation between levels of SIRT2 in serum exosomes and mRS scores. Lastly, a *p*-value of less than 0.05 was deemed to be statistically significant.

## Results

### **An elevated SIRT2 concentration of serum exosomes in acute ischemic stroke patients**

**Fig. 1:**
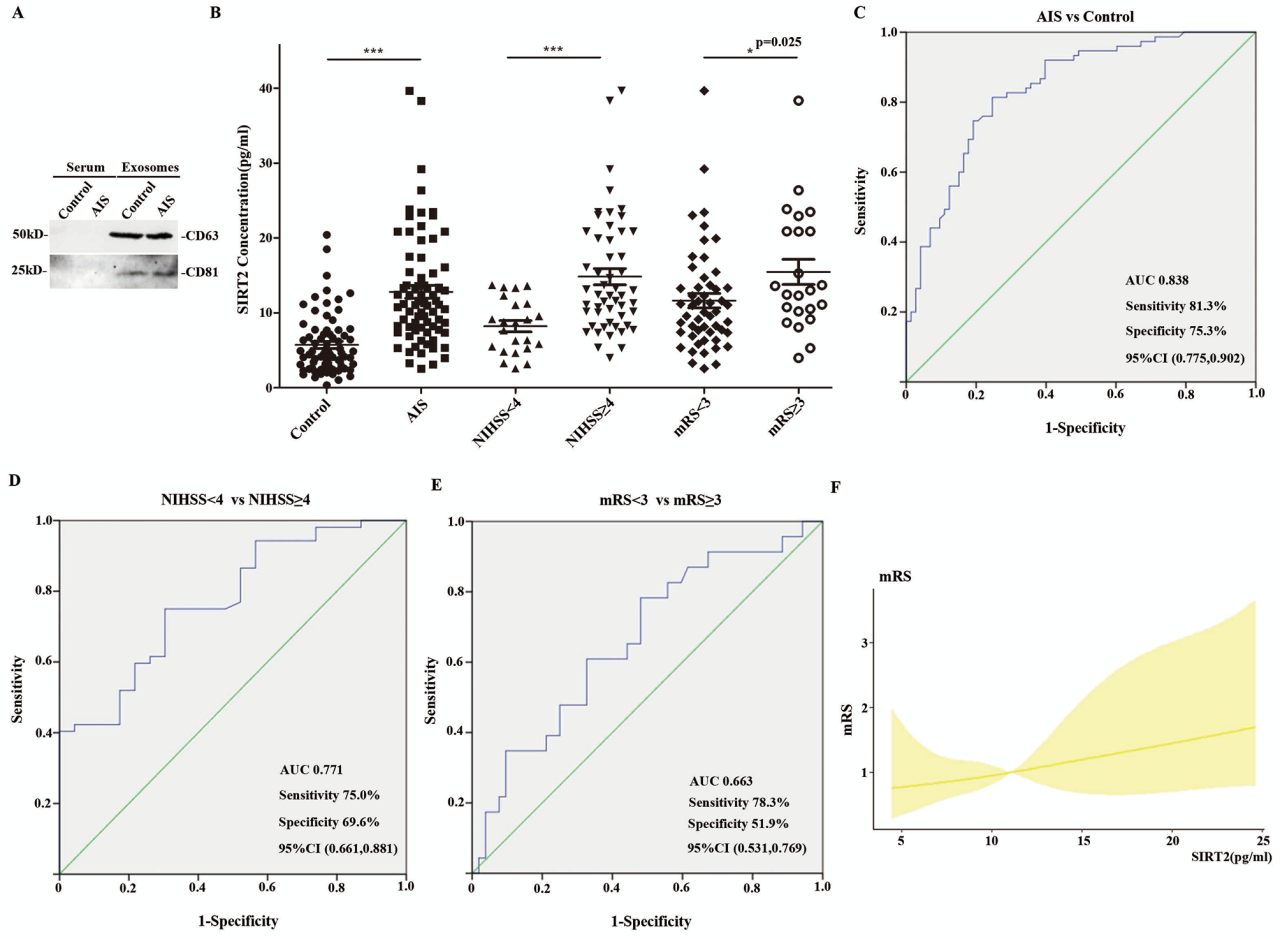
**(A)** Exosomes protein marker of CD63 and CD81 were detected in serum and serum exosomes by western blot. Full-length blots/gels are presented in the Supplementary Fig. 1. **(B)** Serum exosome SIRT2 concentration in control group (n = 75), AIS group (n = 75), NIHSS < 4 (n = 23), NIHSS ≥ 4 (n = 52), mRS < 3 (n = 52), and mRS ≥ 3(n = 23) group. **(C)** Receiver operating characteristic (ROC) curve of the relationship between Serum exosome SIRT2 concentration and acute ischemic stroke. **(D)** Receiver operating characteristic (ROC) curve of the relationship between Serum exosome SIRT2 concentration and NIHSS ≥ 4 acute ischemic stroke. **(E)** Receiver operating characteristic (ROC) curve of the relationship between Serum exosome SIRT2 concentration and mRS ≥ 3 acute ischemic stroke. **(F)** The correlation between serum exosome SIRT2 concentration and mRS score of AIS by restricted cubic spline analysis. *p < 0.05 ***p < 0.001

A total of 150 subjects were finally included in this study, evenly split into two groups. The first group, known as the control group, consisted of 75 individuals with high-risk factors for stroke. Meanwhile, the second group, referred to as the acute ischemic stroke (AIS) group, included 75 patients who were currently experiencing stroke. Although 77 AIS patients were initially enrolled, two were lost to follow-up, leaving a final total of 75 enrolled individuals. To analyze potential biomarkers related to stroke, peripheral blood serum was collected and exosomes were isolated to detect SIRT2. Additionally, two exosomal protein biomarkers, CD63 and CD81, were confirmed in serum exosomes by western blot (WB) analysis (Fig. 1A). The study also considered demographic and baseline clinical characters of the control and AIS groups in Table 1. Interestingly, there were no significant difference in age, drinking history, the prevalence of diabetes, total cholesterol, triglyceride, low-density lipoprotein, creatinine, and uric acid between the two groups. However, the AIS group had a higher proportion of males and smokers, as well as a significantly higher prevalence of hypertension (*p* < 0.05) and fasting blood glucose (FBG) (*p* < 0.001) compared to the control group (Table 1).

**Table 1:** Demographic and clinical characteristics of control subjects and acute ischemic stroke patients

	Control group (n = 75)	AIS group (n = 75)	P
Age (years)	67.95 ± 7.07	68.24 ± 12.86	0.863
Male (%)	26(34.67)	43(57.33)	0.005^*^
Smoker (%)	13(17.33)	24(32.00)	0.037^*^
Drinker (%)	13(17.33)	12(16.00)	0.827
Hypertension (%)	31(41.33)	49(65.33)	0.003^*^
DM (%)	13(17.33)	14(18.67)	0.832
TC (mmol/L)	4.56 ± 0.89	4.35 ± 0.96	0.154
TG (mmol/L)	1.27 ± 0.65	1.57 ± 1.10	0.051
LDL (mmol/L)	2.91 ± 0.84	2.85 ± 0.87	0.651
FBG (mmol/L)	5.07 ± 0.60	6.64 ± 2.61	< 0.001^***^
Cr (mmol/L)	65.12 ± 11.48	70.40 ± 22.17	0.070
UA (mmol/L)	295.37 ± 83.49	311.67 ± 105.48	0.296
SIRT2 (pg/ml)	4.52(2.67,7.43)	11.11(7.76, 15.46)	< 0.001^***^

DM diabetes mellitus, TC total cholesterol, TG triglyceride, LDL low-density lipoprotein, FBG fasting blood glucose, Cr creatinine, UA uric acid, SIRT2 Silent Information Regulator 2. *p < 0.05 ***p < 0.001

As shown in the Mann-Whitney U test, the AIS group had a higher concentration of SIRT2 in serum exosomes compared to the control group (*p* < 0.001) (Fig. 1B). To further investigate the potential of serum exosome SIRT2 as a biomarker for AIS, the receiver operating characteristic (ROC) curve was computed. The ROC analysis showed a positive correlation between higher serum exosome SIRT2 and AIS with a sensitivity of 81.3% and a specificity of 75.3%. The area under the curve was 0.838 (95% CI: 0.775, 0.902), and the cut-off value deemed significant was 7.26 pg/ml, demonstrated in Fig. 1C. Binary Logistic analysis implicated the level of serum exosome SIRT2 as independent risk factors of AIS (odd ratio = 1.394, 95%CI 1.231–1.577, p < 0.001) (Table 2).

**Table 2:** Logistic regression of risk for acute ischemic stroke

	Univariate analysis				Multivariate analysis		
	OR	95% CI	P		OR	95% CI	P
Age (years)	1.003	(0.972,1.034)	0.862				
Male	2.532	(1.309,4.899)	0.006^*^		0.423	(0.067,2.696)	0.363
Smoker	2.244	(1.039,4.847)	0.040^*^		1.678	(0.426,6.603)	0.459
Drinker	0.908	(0.385,2.145)	0.827				
Hypertension	2.675	(1.381,5.182)	0.004^*^		8.543	(1.368,53.329)	0.022^*^
DM	1.095	(0.476,2.519)	0.832				
TC (mmol/L)	0.774	(0.544,1.102)	0.155				
TG (mmol/L)	1.456	(0.987,2.148)	0.058		1.200	(0.632,2.279)	0.577
LDL (mmol/L)	0.916	(0.628,1.335)	0.648				
FBG (mmol/L)	1.928	(1.393,2.669)	< 0.001^***^		2.304	(1.459,3.638)	< 0.001^***^
Cr (mmol/L)	1.020	(0.998,1.042)	0.081		1.013	(0.974,1.053)	0.520
UA (mmol/L)	1.002	(0.998,1.042)	0.296				
SIRT2 (pg/ml)	1.316	(1.192,1.454)	< 0.001^***^		1.394	(1.231,1.577)	< 0.001^***^

Multivariate logistic regression analysis was employed when variables with a *p*-value were less than 0.1. DM diabetes mellitus, TC total cholesterol, TG triglyceride, LDL low-density lipoprotein, FBG fasting blood glucose, Cr creatinine, UA uric acid, SIRT2 Silent Information Regulator 2, OR odds ratio, CI confidence interval. *p < 0.05 ***p < 0.001

### A higher SIRT2 concentration of serum exosomes in AIS patients with higher NIHSS

In order to investigate the correlation between serum exosome SIRT2 and the severity of AIS, AIS patients were separated into two cohorts, one comprising 23 patients with mild stroke (NIHSS < 4), and another consisting of 52 patients with an NIHSS score of ≥ 4. As shown in Table 3, the average age, creatinine and uric acid (*p* < 0.001) were significantly higher in the NIHSS ≥ 4 group when compared to the NIHSS < 4 group. However, there was no difference between the two groups in terms of gender, smoking history, drinking history, the prevalence of hypertension, diabetes, and the level of triglyceride.

**Table 3:** Demographic and clinical characteristics of acute ischemic stroke patients between NIHSS ≥ 4 and NIHSS＜4.

	NIHSS < 4 group (n = 23)	NIHSS ≥ 4 group (n = 52)		P
Age (years)	69.59 ± 10.81	71.08 ± 11.78	< 0.001^***^	
Male (%)	11(47.83)	32(61.54)	0.268	
Smoker (%)	5(21.74)	19(36.54)	0.205	
Drinker (%)	4(17.39)	8 (15.38)	0.827	
Hypertension (%)	14(60.87)	35(67.31)	0.589	
DM (%)	5(21.74)	9(17.31)	0.650	
TC (mmol/L)	4.76 ± 1.06	4.46 ± 0.94	0.011^*^	
TG (mmol/L)	2.23 ± 1.21	2.37 ± 1.55	0.648	
LDL (mmol/L)	3.29 ± 0.92	3.02 ± 0.87	0.042^*^	
FBG (mmol/L)	8.19 ± 3.43	7.40 ± 2.80	0.012^*^	
Cr (mmol/L)	65.00 ± 11.77	81.92 ± 32.43	< 0.001^***^	
UA (mmol/L)	294.76 ± 62.26	366.92 ± 137.26	< 0.001^***^	
SIRT2 (pg/ml)	9.55(6.36,13.34)	17.52(11.54,23.42)	< 0.001^***^	

DM diabetes mellitus, TC total cholesterol, TG triglyceride, LDL low-density lipoprotein, FBG fasting blood glucose, Cr creatinine, UA uric acid, SIRT2 Silent Information Regulator 2. *p < 0.05 ***p < 0.001

As shown in Fig. 1B, it was found that the concentration of SIRT2 in serum exosomes was higher in patients with NIHSS ≥ 4, indicating worse neurological functions (*p* < 0.001). The result of a ROC analysis showed that serum exosomes SIRT2 was able to effectively discriminate between AIS patients with NIHSS ≥ 4 and those with mild stroke (NIHSS < 4), with a sensitivity of 75.0%, a specificity of 69.6%, and an area under the curve of 0.771 (95% confidence interval: 0.661,0.881). The cut-off value was 9.565 pg/ml (Fig. 1D). Binary Logistic analysis showed that the SIRT2 concentration in serum exosomes was an independent risk factor for AIS with NIHSS ≥ 4 (odd ratio = 1.258, 95%CI 1.084–1.460, p = 0.002) **(**Table 4**)**.

**Table 4:** Logistic regression of risk for acute ischemic stroke between NIHSS ≥ 4 and NIHSS＜4.

	Univariate analysis				Multivariate analysis		
	OR	95% CI	P		OR	95% CI	P
Age (years)	1.009	(0.971,1.048)	0.659				
Male	1.745	(0.648,4.701)	0.270				
Smoker	2.073	(0.663,6.483)	0.210				
Drinker	0.864	(0.232,3.218)	0.827				
Hypertension	1.324	(0.478,3.664)	0.590				
DM	0.753	(0.222,2.562)	0.650				
TC (mmol/L)	0.794	(0.476,1.325)	0.378				
TG (mmol/L)	1.062	(0.670,1.683)	0.797				
LDL (mmol/L)	0.802	(0.456,1.412)	0.445				
FBG (mmol/L)	0.950	(0.790,1.143)	0.585				
Cr (mmol/L)	1.038	(1.001,1.077)	0.045^*^		1.027	(0.988,1.067)	0.182
UA (mmol/L)	1.005	(0.999,1.011)	0.114				
SIRT2 (pg/ml)	1.279	(1.103,1.482)	0.001^*^		1.258	(1.084,1.460)	0.002^*^

Multivariate logistic regression analysis was employed when variables with a *p*-value were less than 0.1. DM diabetes mellitus, TC total cholesterol, TG triglyceride, LDL low-density lipoprotein, FBG fasting blood glucose, Cr creatinine, UA uric acid, SIRT2 Silent Information Regulator 2, OR odds ratio, CI confidence interval. *p < 0.05

### A higher SIRT2 concentration of serum exosomes in AIS patients with higher mRS

To determine the relationship between the SIRT2 concentration of serum exosomes and the outcome of stroke patients, the SIRT2 concentration of serum exosomes was compared between two groups separated by the score of modified Rankin Scale (mRS) scores, including 52 patients with mRS < 3 and 23 patients with mRS ≥ 3. Interestingly, significantly higher levels of low-density lipoprotein(*p* < 0.05), age, uric acid, and NIHSS score (*p* < 0.001) were detected in the mRS ≥ 3 group compared to the group with mRS < 3. In contrast, no significant differences were found between the two groups with regard to smoking history, drinking history, the prevalence of hypertension and diabetes, total cholesterol, triglyceride, and the fasting blood glucose (Table 5).

**Table 5:** Demographic and clinical characteristics of acute ischemic stroke patients between mRS ≥ 3 and mRS＜3.

	mRS < 3 group (n = 52)	mRS ≥ 3 group (n = 23)	P
Age (years)	68.06 ± 11.32	75.76 ± 10.08	< 0.001^***^
Male (%)	26(50.00)	17(73.91)	0.045^*^
Smoker (%)	14(26.92)	10(43.48)	0.156
Drinker (%)	7(13.46)	5(21.74)	0.367
Hypertension (%)	33(63.46)	16(69.57)	0.609
DM (%)	10(19.23)	4(17.39)	0.850
TC (mmol/L)	4.52 ± 0.99	4.65 ± 0.98	0.281
TG (mmol/L)	2.45 ± 1.49	2.01 ± 1.33	0.146
LDL (mmol/L)	3.02 ± 0.88	3.29 ± 0.90	0.037^*^
FBG (mmol/L)	7.64 ± 2.95	7.68 ± 3.21	0.879
Cr (mmol/L)	78.30 ± 34.58	75.12 ± 11.42	< 0.001^***^
UA (mmol/L)	344.47 ± 132.12	352.12 ± 109.33	< 0.001^***^
NIHSS	8(4, 11)	12(7, 13)	< 0.001^***^
SIRT2 (pg/ml)	13.28(9.55,19.94)	20.88 (12.32,23.63)	0.025^*^

DM diabetes mellitus, TC total cholesterol, TG triglyceride, LDL low-density lipoprotein, FBG fasting blood glucose, Cr creatinine, UA uric acid, NIHSS National Institute of Health Stroke Scale, SIRT2 Silent Information Regulator 2. *p < 0.05 ***p < 0.001

The study found that patients with a higher mRS score had a significantly higher concentration of SIRT2 in their serum exosomes than those with a lower mRS score (*p* < 0.05) (Fig. 1B). ROC analysis revealed the sensitivity, specificity, and the area under the curve for SIRT2 to discriminate AIS with mRS ≥ 3 from AIS with mRS < 3 was 78.3%, 51.9%, and 0.663 (95% CI: 0.531,0.769). The optimal cut-off value for SIRT2 was 10.195 pg/ml (Fig. 1E). Restricted cubic splines analysis confirmed that a higher serum exosome SIRT2 concentration was commonly detected in patients with a higher mRS score (Fig. 1F). However, Binary Logistic analysis did not identify the SIRT2 concentration of serum exosomes was as an independent risk factor for a higher mRS score (odd ratio = 1.065, 95%CI 0.983–1.154, p = 0.125) (Table 6).

**Table 6:** Logistic regression of risk for acute ischemic stroke between mRS ≥ 3 and mRS＜3.

	Univariate analysis				Multivariate analysis		
	OR	95% CI	P		OR	95% CI	P
Age (years)	1.072	(1.016,1.130)	0.011^*^		1.059	(0.991,1.132)	0.093
Male	2.833	(0.964,8.325)	0.058		39.887	(3.973,400.469)	0.002^*^
Smoker	2.088	(0.747,5.832)	0.160				
Drinker	1.786	(0.501,6.366)	0.371				
Hypertension	1.316	(0.459,3.770)	0.609				
DM	0.884	(0.246,3.180)	0.851				
TC (mmol/L)	1/191	(0.715,1.984)	0.501				
TG (mmol/L)	0.828	(0.505,1.356)	0.453				
LDL (mmol/L)	1.511	(0.850,2.687)	0.160				
FBG (mmol/L)	1.016	(0.843,1.226)	0.865				
Cr (mmol/L)	1.008	(0.986,1.029)	0.483				
UA (mmol/L)	1.001	(0.997,1.006)	0.608				
NIHSS	1.214	(1.071,1.376)	0.002^*^		1.471	(1.171,1.848)	0.001^*^
SIRT2 (pg/ml)	1.071	(1.000,1.146)	0.049^*^		1.065	(0.983,1.154)	0.125

Multivariate logistic regression analysis was employed when variables with a *p*-value were less than 0.1. DM diabetes mellitus, TC total cholesterol, TG triglyceride, LDL low-density lipoprotein, FBG fasting blood glucose, Cr creatinine, UA uric acid, NIHSS National Institute of Health Stroke Scale, SIRT2 Silent Information Regulator 2, OR odds ratio, CI confidence interval. *p < 0.05

## Discussion

Occlusion of the cerebral artery generally leads to cerebral infarction, which caused heavy financial and medical burden. Ischemia and reperfusion (IR), which occur in acute ischemic stroke (AIS), can result in various detrimental effects such as neuroinflammation, peroxidation, and increased permeability of the vessels, apoptosis, necrosis, and blood-brain barrier (BBB) injury [22–24]. Recently, a number of studies have shown that SIRT2, may have an association with stroke. In animal models, it was observed that SIRT2 mRNA was upregulated after cerebral ischemia, and by knocking out SIRT2, neurological functions were preserved [25]. Furthermore, SIRT2 inhibitors, such as AK1 or AGK2, have been reported to reduce cell apoptosis by downregulating the AKT/FOXO3a and MAPK pathways, resulting in a reduction in the ipsilateral infarct area after ischemic stroke [26]. In addition, in a study conducted on mice, a SIRT2 inhibitor called AK7 was found to decrease the infarct size and attenuate neurological deficits by activating the P38 MAPK pathway [16].These findings support the results of the research study, which revealed that a higher concentration of SIRT2 in serum exosomes was associated with more severe NIHSS scores. Notably, recently reports have shown that SIRT2 protein is upregulated in the brain tissue of a mouse model with transient middle cerebral artery occlusion [27].The overexpression of SIRT2 protein after ischemic stroke may have contributed to the increased SIRT2 concentration in AIS serum exosomes which was found in this study. By exploring the relationship between SIRT2 and clinical symptoms, a better understanding of the mechanism through which SIRT2 plays a role in ischemic stroke can be gained.

Effective blood biomarkers are highly valued in the medical community because they are easy to collect and non-invasive, making them better for clinical decisions. Recently, there have been some studies on the use of blood biomarkers in diagnosing central nervous system disease. In particular, SIRT2 has been shown as a potential blood biomarker to differentiate Parkinson’s disease from Parkinsonian Syndromes. Researchers discovered that elevated serum SIRT2 protein was correlated with higher α-Syn Parkinson’s disease patients [17]. Fortunately, SIRT2 could deacetylate α-synuclein, it can have a neuroprotective effect [14, 28]. In addition, studies have also shown that elevated serum SIRT2 is present in acute ischemic stroke patients. Higher levels of serum SIRT2 are associated with overexpression of inflammatory factors in AIS patients with severe neurological deficits [29]. However, it is important to remember that blood biomarkers could not reflect the severity of neurological disorders accurately. That’s why cerebrospinal fluid is considered a more superior sample for diagnosing and treating central nervous system diseases. Unfortunately, cerebrospinal fluid collection is not always practical in clinical settings because it can be painful and invasive. As a result, researchers have started exploring other sources of biomarkers, including serum exosomes. Exosomes are tiny structures that are capable of crossing blood-brain barrier with high delivery efficient and low immunogenicity. They may be a useful tool for diagnosing and predicting the course of central nervous system conditions [30].

Exosomes are a class of granular subcellular organelles secreted by cells that contain signaling molecules, such as mRNAs, microRNAs, and proteins. These signaling molecules are known to play important roles in mediating communications between cells [21, 31]. In the central nervous system, cells like oligodendrocytes and neurons also secrete exosomes which carry specific proteins and RNA cargos [32]. This communication between neurons and glial cells via exosomes is critical for maintaining the integrity of the nervous system [33]. What’s more, exosomes mediate interactions between astrocytes and other brain cells [20, 34]. Astrocyte-derived exosomes have been proved to ameliorate neuronal damage in ischemic stroke by suppressing apoptosis and inflammation [35]. Central nervous system derived exosomes were detected in the cerebrospinal fluid and peripheral body fluids because exosomes can penetrate the blood-brain barrier (BBB) and are highly stable in circulating blood [36]. These results suggest that serum exosomes may be a potential source of biomarkers for diagnosis and management of CNS diseases. In fact, our study found that SIRT2, a protein found in serum exosomes, may be a potential biomarker for the diagnosis of AIS. We observed a positive correlation between enhanced exosomes SIRT2 level and both NIHSS and mRS scores, which are commonly used metrics for assessing stroke severity and functional disability. These findings suggest that detection of the concentration of SIRT2 in serum exosomes could facilitate diagnosis and prediction of patient outcomes in stroke. Moreover, inhibiting SIRT2 in serum exosomes could potentially be a therapeutic strategy to protect against the harmful effects of stroke. Our future study will comprehensively explore the influence of serum exosome SIRT2 on AIS in mice and further elucidate the underlying pathophysiological mechanism.

Limitations of the study.

The limitations of the study include the small sample size, and the low SIRT2 concentration of serum exosomes may weaken the ability to establish a strong correlation between serum exosomes SIRT2 levels and acute ischemic stroke.

## Conclusions

To summarize, this study indicates that the SIRT2 concentration in serum exosomes is elevated after acute ischemic stroke has occurred. Patients with a higher level of serum exosome SIRT2 also tend to have higher NIHSS scores, meaning that they experience more neurological deficits, and have higher mRS scores, indicating a poorer functional outcome. The research demonstrates that high levels of serum exosome SIRT2 serve as an independent risk factor for diagnosing AIS and higher NIHSS scores (≥ 4). Overall, these findings provide valuable insights into the role of serum exosome SIRT2 in the pathogenesis of AIS and highlights the importance of identifying novel biomarkers for improving diagnosis and treatment of stroke.

### Electronic supplementary material

Below is the link to the electronic supplementary material.

Supplementary Material 1

Supplementary Material 2

Supplementary Material 3

## Data Availability

All data generated or analyzed during this study are included in the supplementary information files.

## Abbreviations

SIRT2: Silent Information Regulator 2
AIS: Acute ischemic stroke
NIHSS: National Institute of Health Stroke Scale
mRS: The modified Rankin Scale score
ROC: Receiver operating characteristic curve
BBB: Blood-brain barrier
ELISA: Enzyme linked immunosorbent assay
WB: Western blot
