# Supplementary figures and images for: Elevated SIRT2 of serum exosomes is positively correlated with diagnosis of acute ischemic stroke patients
Source: BMC Neurol. 2023 Sep 8;23:321. doi: 10.1186/s12883-023-03348-7 (PMC10485972; doi:10.1186/s12883-023-03348-7)

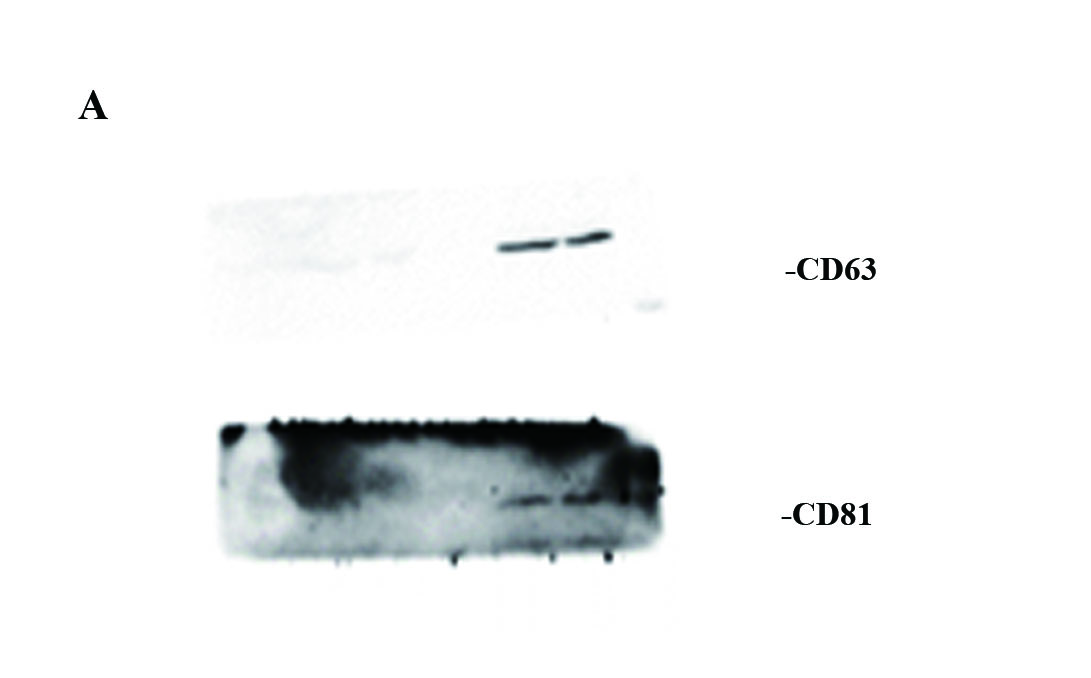

Supplement: Supplementary file 2 — Supplementary Material 2 [file 12883_2023_3348_MOESM2_ESM.jpg]
