# Supplementary material for: Elevated SIRT2 of serum exosomes is positively correlated with diagnosis of acute ischemic stroke patients
Source: BMC Neurol. 2023 Sep 8;23:321. doi: 10.1186/s12883-023-03348-7 (PMC10485972; doi:10.1186/s12883-023-03348-7)
Supplement: Supplementary file 3 — Supplementary Material 3 [file 12883_2023_3348_MOESM3_ESM.docx]

**Supplementary Figure1.** (A) Full-length blots/gels of CD63 and CD81.
